# Phylogeographic Analyses Reveal a Crucial Role of Xinjiang in HIV-1 CRF07_BC and HCV 3a Transmissions in Asia

**DOI:** 10.1371/journal.pone.0023347

**Published:** 2011-08-12

**Authors:** Jun Liu, Chiyu Zhang

**Affiliations:** Institute of Life Sciences, Jiangsu University, Zhenjiang, Jiangsu, China; Institut Pasteur, France

## Abstract

**Background:**

China faces an increasing prevalence of two HIV-1 circulating recombinant forms (CRFs) 07_BC and 08_BC. Both CRFs_BC were previously demonstrated to originate in Yunnan and spread to Liaoning from Yunnan via injection drug use (IDU) in China. Supposing it is true, we are unable to answer why only CRF07_BC, rather than both CRFs_BC together, was transmitted to Xinjiang.

**Methodology/Principal Findings:**

We investigated the phylogeography of CRF07_BC and CRF08_BC using multiple HIV-1 genomic regions with Bayesian phylogeography method. Phylogenetic reconstructions showed that all CRF07_BC sequences were divided into two clades, Yunnan and Xinjiang, and all strains from other regions of mainland China clustered within the Xinjiang clade. Significant geographic diffusion links of Xinjiang with other regions (including Liaoning, Beijing, Jiangsu and Guangdong) were supported by Bayes factor tests. The temporal dynamics analyses showed that CRF07_BC spread from Xinjiang to Liaoning in 1996.10, and to Jiangsu in 2000.9. The analyses of CRF08_BC not only confirmed the previous conclusion on temporal and spatial dynamics of CRF08_BC, but also indicated that the CRF08_BC strains from Guangdong and Shanghai originated from Yunnan. The analyses of HCV 3a showed that it was introduced into Xinjiang in the early 1980s, and spread from Xinjiang to Yunnan in 1990.10 and to Jiangsu in 1999.2, and further from Yunnan to Guangxi in 1995.3. The temporal and spatial dynamics of HCV 3a were similar to some extent to that of HIV-1 CRF07_BC and/or CRF08_BC, suggesting a possible association in migration patterns between HCV and HIV-1 through IDU. In addition, HCV 3a spread from Xinjiang to Pakistan, implying a drug trafficking route linking them.

**Conclusions/Significance:**

Xinjiang, as the most important transfer station for drug trafficking from Golden Crescent to other regions of China, plays a very crucial role in the transmission of viruses (e.g., HIV-1 and HCV) through IDU in Asia.

## Introduction

Yunnan Province of China borders the ‘Golden Triangle’ region (one of the world's largest heroin-producing areas) and is the most important epicenter for heroin smuggling into China and other Asia countries or regions [1], [2]. Because of the special location of Yunnan, human immunodeficiency virus type 1 (HIV-1) subtypes B' (Thai-B subtype) and C that originated in Thailand and India, respectively, were introduced into Yunnan via drug-trafficking routes and resulted in the initial HIV-1 outbreak among injection drug users (IDUs) in Yunnan in the late 1980s [3], [4], [5]. The co-circulation of both subtypes further resulted in the on-going generation of HIV-1 B'/C intersubtype recombinants (referred to as unique recombinant form (URF) or circulating recombinant form (CRF)) in Yunnan [6], [7], [8]. For example, several new B'/C recombinants were identified in southwestern region (Dehong Prefecture) of Yunnan that borders Myanmar [6], [7], [8]. HIV-1 CRF07_BC and CRF08_BC are the descendents of Thai subtype B' and India subtype C [9], [10]. Both of them were originally found among IDUs in Xinjiang and Guangxi, China, in 1997, respectively [9], [10]. Since then, both CRF07_BC and CRF08_BC were demonstrated to be predominant strains co-circulating in Yunnan [6], [8]. Based on these observations, both CRFs_BC were naturally thought to originate in Yunnan.

To address the hypothesis that HIV-1 CRF07_BC and CRF08_BC originated in Yunnan, Tee et al. (2008) investigated the temporal and spatial dynamics of both CRFs_BC across East Asia [11], [12], [13]. Based on the estimation of the time to the most recent common ancestor (tMRCA) and the reconstruction of ancestral relationships, they clearly confirmed the migration pattern of CRF08_BC, in which CRF08_BC originated in Yunnan in 1990 and further spread northeastward to Liaoning and eastward to Guangxi around 1995. In addition, they inferred that CRF07_BC also originated in Yunnan in 1993, and further concluded that the migration of CRF07_BC from Yunnan to Xinjiang (northwest), Liaoning (northeast), and Taiwan (east) were via different drug trafficking routes [11], [12].

The origin and migration route of CRF07_BC, however, still remain elusive. If the previous conclusions on the migration routes of CRF07_BC and CRF08_BC described by Tee et al. [11], [12] are correct, there remain two questions that cannot be answered objectively. Firstly, both CRFs_BC could be transmitted northeastward to Liaoning from Yunnan. However, why was only CRF07_BC transmitted northwestward to Xinjiang, but not CRF08_BC? Secondly, CRF08_BC originated in Yunnan in 1990, earlier than CRF07_BC. Until 1995, CRF08_BC started to spread to Guangxi (the neighboring province of Yunnan) and Liaoning. However, why CRF07_BC was able to spread rapidly to the regions (Xinjiang and Liaoning) far away from Yunnan in the same year (1993), shortly after when it originated in 1993?

Bayesian phylogeography method, a probabilistic method developed recently by Lemey et al., is more capable of describing the most plausible scenario of geographic migration than the previous methods [14]. To address this issue, here we used the Bayesian phylogeography method to revisit the origin and phylogeography of HIV-1 CRF07_BC and CRF08_BC based on multiple HIV-1 genomic regions with more tip-dated sequences. Our results show that CRF07_BC spread from Xinjiang, rather than Yunnan, to other regions of mainland China, and the geographic origin of CRF07_BC is more complex than previously thought. In addition, additional analysis shows that hepatitis C virus (HCV) 3a also spread from Xinjiang to other regions of China and even to Pakistan. These results indicate that Xinjiang plays a very crucial role in transmission of some viruses through injection drug use in Asia.

## Results

HIV-1 CRF07_BC and CRF08_BC have subtype C backbones with several insertions of subtype B'. In previous studies, only one fragment of subtype C origin was selected to investigate the spatial and temporal spread of CRF07_BC and CRF08_BC [11], [12]. In order to obtain overall insight into the origin and migration of CRF07_BC and CRF08_BC in Asia, here we selected multiple non-recombinant genomic regions of subtype C or B' origin, located in *gag*, *gag-pol* and *env* gene regions, to perform phylogeographic analyses. Two subtype C-originated fragments in *gag* region of CRF07_BC (HXB2 793-1211 nt and 1462-2061 nt) are separated by a short subtype B' insertion. Since more informative sites in analyzed sequences can enhance the accuracy of phylogenetic analysis, we performed the partition homogeneity test using PAUP* [15] to evaluate whether two split subtype C fragments (HXB2 793-1211 nt and 1462-2061 nt) can be merged into one continuous sequence. The results show that the two split fragments of CRF07_BC were homogeneous (p = 0.64). A similar result (p = 0.57) was also observed in two fragments (HXB2 794-1209 nt and 1709-2826 nt) of CRF08_BC. These results indicate that the two subtype C fragments in CRF07_BC or CRF08_BC can be merged into one fragment in phylogenetic analyses.

### Phylogeography of HIV-1 CRF07_BC

**Figure 1:**
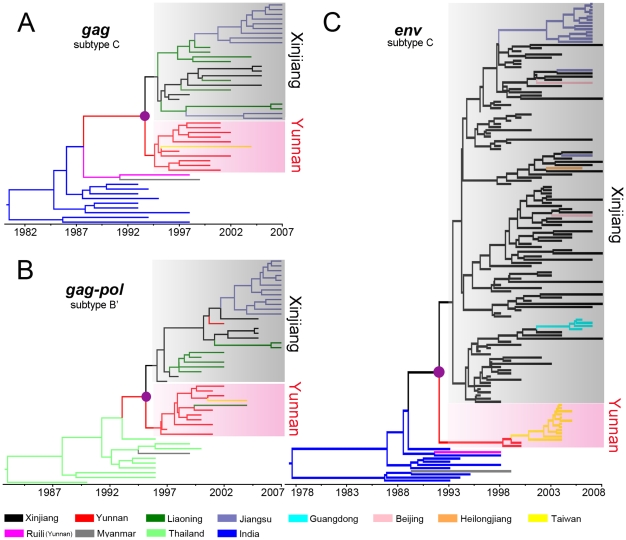
Maximum clade credibility trees of the HIV-1 CRF07_BC sequences. Ancestral geographic states were reconstructed using Bayesian phylogeographic inference framework implemented in the BEAST v1.5.4 package. The tree branches are colored according to their respective geographical locations. The purple solid nodes on the trees represent the most recent common ancestor (MRCA) of CRF07_BC. (A) The maximum clade credibility (MCC) tree reconstructed based on the merged *gag* region (HXB2 793-1211 nt and 1462-2061 nt) of subtype C origin. (B) The MCC tree reconstructed based on the *gag-pol* region (HXB2 2102-2601 nt) of subtype B' origin. (C) The MCC tree reconstructed based on the *env* region (HXB2 7095-7328 nt) of subtype C origin.

Phylogenetic reconstruction of the merged *gag* region of subtype C origin (Fig. 1**A**) shows that all CRF07_BC sequences were divided into two clades. All strains (red) from Yunnan clustered together, forming the Yunnan clade. Except Yunnan strains, only one strain from Taiwan was included in this clade, indicating a close genetic relationship between Yunnan and Taiwan strains. Intriguingly, all strains from other regions of mainland China, including Xinjiang, Liaoning and Jiangsu, clustered together and formed another clade with a root in Xinjiang (black). It indicates that the strains from at least Liaoning and Jiangsu have close evolutionary associations with Xinjiang strains. To test whether there are some statistically significant geographic diffusion links (BF>3) between these strains, the Bayes factor (BF) test was performed. Three significant geographic links were clearly observed between Xinjiang and Liaoning (BF = 234.944), between Liaoning and Jiangsu (BF = 18.592), and between Yunnan and Taiwan (BF = 7.040) (Table 1). These results strongly argue against the previous conclusion on the migration route of CRF07_BC from Yunnan to Xinjiang and Liaoning.

**Table 1:** The Bayes factors between defined locations of the CRF07_BC *gag-pol* region of subtype B' origin (above diagonal) and merged *gag* region of subtype C origin (below diagonal).

Origin	Xinjiang	Jiangsu	Liaoning	Yunnan	Taiwan
**Xinjiang**		2.673	**28.695**	**7.209**	0.739
**Jiangsu**	0.574		0.751	0.655	0.330
**Liaoning**	**234.944**	**18.592**		1.822	0.492
**Yunnan**	2.605	0.141	2.329		**5.619**
**Taiwan**	0.531	0.198	0.431	**7.040**	

Bayes factors above 3 that represent statistically significant phylogeographic links between defined locations are shown in bold.

The genome of HIV-1 CRF07_BC contains some inserted fragments of subtype B'. Like the subtype C fragments, the subtype B' fragments should also retain the evolutionary characteristics reflecting the migration routes of CRF07_BC. Therefore, we extended our analyses to the *gag-pol* region of subtype B' origin (Fig. 1**B**). Similar to the results of subtype C-originated fragment, two clades, the Xinjiang and Yunnan clades, were also observed in the tree of the subtype B' fragment of CRF07_BC. Except one Liaoning strain that clustered within the Yunnan clade, all Liaoning and Jiangsu strains clustered closely within the Xinjiang clade, supporting the close evolutionary associations of the Liaoning and Jiangsu strains with the Xinjiang strains. Further BF test shows that geographic diffusion links between Xinjiang and Liaoning (BF = 28.695), between Xinjiang and Yunnan (BF = 7.209), and between Yunnan and Taiwan (BF = 5.619) are statistically significant (Table 1).

The results of both subtype B' and C fragments in *gag-pol* region clearly indicate that the CRF07_BC strains circulating in Liaoning and Jiangsu originated from Xinjiang, rather than from Yunnan. Because of the limited *gag-pol* sequences available for CRF07_BC, however, it is unclear whether CRF07_BC from other regions of mainland China also originated from Xinjiang. Since a great deal of C2V3 sequences were available for a relatively wide range of locations across mainland China, to address this issue, we further extended our analyses to the C2V3 fragment in *env* region of CRF07_BC. These locations cover Xinjiang, Yunnan, Jiangsu, Beijing, Guangdong, Heilongjiang, and Taiwan.

Phylogenetic reconstruction of the *env* region shows that the CRF07_BC sequences were still divided into two clades of Xinjiang and Yunnan. All strains from Taiwan still clustered with the Yunnan strains, firmly confirming the migration route of CRF07_BC from Yunnan to Taiwan. Except the strains from Yunnan and Taiwan, all strains from other regions of mainland China clustered within the Xinjiang clade and were rooted with the Xinjiang strains (Fig. 1**C**). The BF values show statistically significant geographic diffusion links between Xinjiang and Jiangsu (BF = 280.206), and Beijing (BF = 25.129), and between Yunnan and Taiwan (BF = 34.702) (Table 2). In this analysis, only 2 Yunnan sequences were isolated in and before 2000, whereas 34 Xinjiang sequences were isolated before 2000. Because the use of more early CRF07_BC sequences from Xinjiang than from Yunnan may lead to a bias in the ancestral state inference of CRF07_BC strains in phylogeographic analysis, we excluded 34 Xinjiang sequences before 2000 and re-performed the analysis. The results are consistent with that obtained using all sequences of *env* fragment (Fig. S1 **A** and Table 2), supporting above observation.

**Table 2:** The Bayes factors between defined locations of the CRF07_BC *env* region of subtype C origin.

Origin	Yunnan	Beijing	Guangdong	Heilongjiang	Taiwan	Xinjiang	Jiangsu
**Yunnan**		1.500	0.821	0.917	**22.575**	1.681	0.496
**Beijing**	1.406		1.764	1.400	0.508	**24.355**	0.498
**Guangdong**	0.770	1.764		0.804	0.371	**3.026**	0.807
**Heilongjiang**	0.918	1.708	0.910		0.413	1.254	0.585
**Taiwan**	**34.702**	0.432	0.359	0.383		0.212	0.229
**Xinjiang**	1.210	**25.129**	2.914	1.930	0.167		**297.133**
**Jiangsu**	0.534	0.520	0.894	0.705	0.238	**280.206**	

Above diagonal: The dataset including CRF07_BC sequences isolated after 2000; below diagonal: the dataset including all available CRF07_BC sequences. Bayes factors above 3 that represent statistically significant phylogeographic links between defined locations are shown in bold.

All above results not only strongly indicate that unlike previously thought, CRF07_BC spread to other regions of mainland China from Xinjiang, rather than from Yunnan, but also imply that Xinjiang plays a more important role than Yunnan at least in the spatial dynamics of CRF07_BC in mainland China.

### Phylogeography of HIV-1 CRF08_BC

HIV-1 CRF07_BC and CRF08_BC are genetically closely related and were considered to be derived from a putative common ancestral B'/C recombinant occurring in Ruili city of Yunnan [7], [16]. In order to test whether the migration route of CRF08_BC changed as that of CRF07_BC, we extended phylogeographic analyses to CRF08_BC datasets, which cover the sequences from more regions of China.

**Figure 2:**
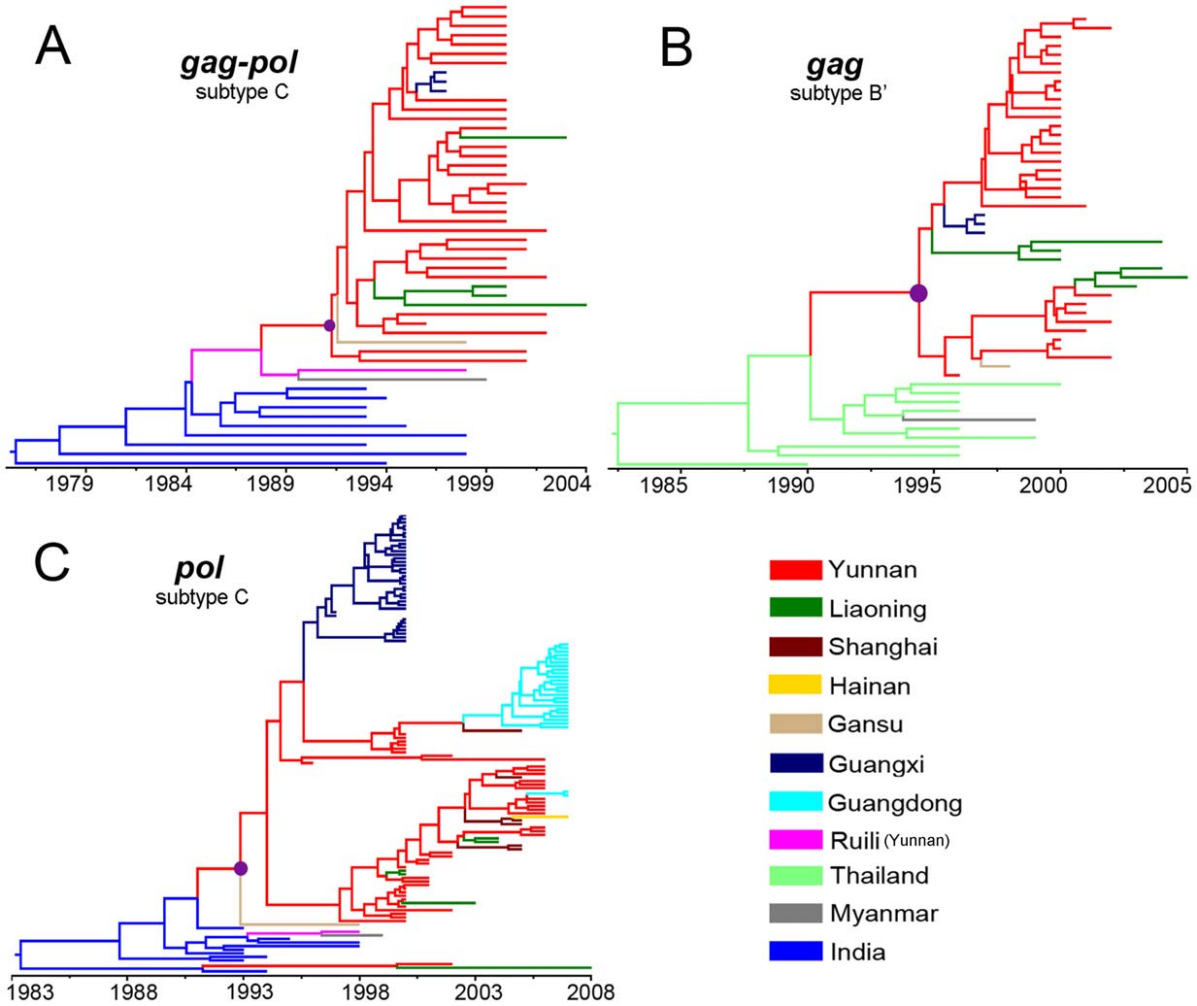
MCC trees of the HIV-1 CRF08_BC sequences. (A) The MCC tree reconstructed based on the merged *gag-pol* region (HXB2 794-1209 nt and 1709-2826 nt) of subtype C origin. (B) The MCC tree reconstructed based on the CRF08_BC *gag* region (HXB2 1258-1658 nt) of subtype B' origin. (C) The MCC tree reconstructed based on the *pol* region (HXB2 2452-2844 nt) of subtype C origin. For other details, see Figure 1.

The MCC trees constructed based on the *gag-pol* region of subtype C origin (Fig. 2**A**) and the *gag* region of subtype B' origin (Fig. 2**B**) consistently show that all CRF08_BC sequences were grouped in a single clade with high posterior probabilities (PP = 1.00). The strains from Guangxi, Liaoning, and Gansu were rooted with the Yunnan strains. The BF tests show the geographic diffusion of CRF08_BC in Guangxi, Liaoning, and Gansu were significantly linked with Yunnan (BF>3) (Table 3). These results clearly indicate that CRF08_BC spread to Guangxi and Liaoning from Yunnan.

**Table 3:** The Bayes factors between defined locations of the CRF08_BC *gag* region of subtype B' (above diagonal) and merged *gag-pol* region of subtype C (below diagonal).

Origin	Yunnan	Gansu	Guangxi	Liaoning
**Yunnan**		**5.529**	**7.425**	**34.715**
**Gansu**	1.868		0.968	0.545
**Guangxi**	**14.175**	1.328		0.627
**Liaoning**	**12.901**	1.103	0.783	

Bayes factors above 3 that represent statistically significant phylogeographic links between defined locations are shown in bold.

Because a large number of the subtype C fragments in *pol* region of CRF08_BC were available, we reconstructed the MCC tree of the *pol* fragment of subtype C origin (Fig. 2**C**). Consistent with the previous results, except that two isolates from Yunnan and Liaoning were located at the base, along with a Indian subtype C strain, all CRF08_BC strains from China (including Guangxi, Guangdong, Liaoning, Shanghai, and Hainan) clustered within the Yunnan group. In addition, the MCC tree of C2V3 fragment in *env* region of CRF08_BC also show that all China strains clustered within the Yunnan group (Fig. S1 **B**). The BF values show that the locations of Guangxi, Liaoning, Guangdong, Shanghai and Hainan had statistically significant geographic diffusion links (BF>3) with Yunnan (Table 4). These results suggest that besides Liaoning and Guangxi, other regions of China, such as Gansu, Guangdong, Shanghai and Hainan, also have significant geographic diffusion links with Yunnan in the migration of CRF08_BC.

**Table 4:** The Bayes factors between defined locations of the CRF08_BC *env* region (above diagonal) and *pol* region (below diagonal) of subtype C.

Origin	Yunnan	Guangxi	Gansu	Hainan	Liaoning	Shanghai
**Yunnan**		**>42754.267**	0.970	**3.277**		
**Guangxi**	**15.463**		2.825	0.555		
**Gansu**	0.594	0.300		1.167		
**Hainan**	1.111	0.264	0.935			
**Liaoning**	**61.277**	0.518	1.484	1.162		
**Shanghai**	**29.340**	0.437	1.095	**3.406**	1.332	
**Guangdong**	**5.113**	0.417	0.215	0.391	0.508	1.677

Bayes factors above 3 that represent statistically significant phylogeographic links between defined locations are shown in bold.

### Origin of HIV-1 CRF07_BC and CRF08_BC

**Figure 3:**
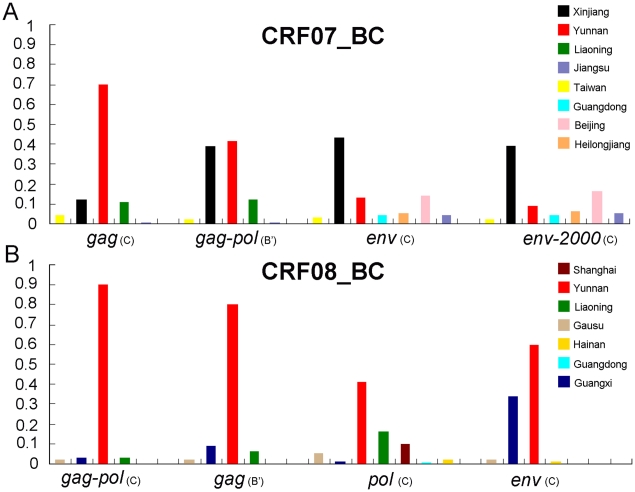
The root state posterior probabilities of CRF07_BC and CRF08_BC based on multiple genomic regions. The subtype origin of each genomic region is shown in the parenthesis. (A) The root state posterior probabilities of CRF07_BC based on the genomic regions of *gag*, *gag-pol* and *env*. The dataset named *env-2000* includes the *env* sequences isolated in China in and after 2000. (B) The root state posterior probabilities of CRF08_BC based on the genomic regions of *gag-pol, gag, pol* and *env*.

Although we demonstrated that CRF07_BC spread to other regions of mainland China from Xinjiang, the geographic origin of CRF07_BC still remains elusive. In ancestral reconstructions, the subtype C fragment in *gag* region and the subtype B' fragment in *gag-pol* region show that the geographic origin of CRF07_BC was Yunnan (Fig. 1**A and B**), whereas the subtype C fragment in *env* region shows that the geographic origin was Xinjiang (Fig. 1**C** and S1**A**). The discordance in the ancestral reconstructions of CRF07_BC between different genomic regions is also reflected by the root state posterior probabilities for the locations (Fig. 3**A**). These results at least suggest that the CRF07_BC strains circulating in China are unlikely to simply derive from a common ancestor that existed in Yunnan in 1993.

Different from CRF07_BC, all ancestral state reconstructions of four analyzed CRF08_BC fragments show that the geographic origin of the CRF08_BC strains circulating in China were pointed toward Yunnan (Fig. 3**B**). These results firmly confirm the previous conclusion that CRF08_BC originated in Yunnan.

### Evolutionary rates and temporal Spread of HIV-1 CRF07_BC and CRF08_BC

We used BEAST package to estimate the evolutionary rates and the dates of tMRCA of HIV-1 CRF07_BC and CRF08_BC. The results show that the evolutionary rates of different genomic regions of CRF07_BC or CRF08_BC were various (Table 5). Since *env* gene is the most hypervariable region of the HIV-1 genome, the *env* fragments (HXB2 7095-7328n) of both CRF07_BC and CRF08_BC unsurprisingly exhibited the highest evolutionary rates [17]. In addition, the evolutionary rates of the subtype B' fragments were higher than those of the merged subtype C fragments. The dates of tMRCA inferred based on different fragments also appear to be different. For example, the dates of tMRCA inferred for CRF07_BC and CRF08_BC based on the subtype B' fragments were later than those based on the merged subtype C fragments. The difference in the dates of tMRCA may be ascribed to the intersubtype recombination.

**Table 5:** Estimated timescale of HIV-1 CRF07_BC, CRF08_BC and HCV 3a

Strain and Fragment a	Genomic Region b	Date of tMRCA c	Rate of evolution d
**CRF07_BC**			
* gag* (C)	793-1211 &1462-2061	1993.8 (1991.9-1995.4)	2.953 (2.192-3.784)
* gag-pol* (B')	2102-2601	1995.1 (1993.5-1996.6)	4.722 (2.898-6.674)
* env* (C)	7095-7328	1991.2 (1988.10-1994.7)	13.537 (10.722-16.365)
**CRF08_BC**			
* gag-pol* (C)	794-1209 &1709-2826	1991.3 (1988.6-1993.4)	2.064 (1.5-2.589)
* gag* (B')	1258-1658	1994.4 (1992.8-1995.11)	3.796 (2.059-5.744)
* env* (C)	7095-7328	1993.5 (1991.2-1995.8)	19.664 (13.293-27.150)
**HCV 3a**			
* E1/E2*	1356-1826	1981.3 (1973.1-1988.6)	7.157 (4.338-9.546)

a The corresponding origin subtype of each genomic region are shown in the parenthesis.

b The genomic regions of CRF07_BC and CRF08_BC were located based on HXB2 genome. The genomic region HCV 3a was located based on H77 genome. The unit is nt (nucleotide).

c The 95% highest posterior density (HPD) credible regions are given in parentheses.

d Rates of evolution are expressed as 10^−3^ nucleotide substitutions per site per year. The 95% credible regions are given in the parentheses.

Based on the merged subtype C fragments, the tMRCA of HIV-1 CRF07_BC and CRF08_BC were estimated at 1993.8 (95% credible region (CR), 1991.9 to 1995.4) and 1991.3 (95% CR, 1988.6 to 1993.4), respectively, similar to the previous estimations (Table 5) [11], [12]. The temporal and spatial dynamics show that after generation from Yunnan in 1991.3, HIV-1 CRF08_BC was disseminated into Liaoning in 1994.11 (95% CR, 1992.8-1997.4) and Guangxi in 1996.2 (95% CR, 1995.7-1996.9). The result of CRF08_BC is similar to the previous studies. For HIV-1 CRF07_BC, although unable to determine from where, Yunnan or Xinjiang, it originated, we demonstrated that after the generation in 1993.8, it spread to Liaoning from Xinjiang in 1996.10 (95% CR, 1994.11-1998.8), and was subsequently disseminated into Jiangsu from Liaoning in 2000.9 (95% CR, 1998.10-2002.7).

### Phylogeography and temporal dynamics of HCV 3a

The transmissions of HIV-1 CRF07_BC and CRF08_BC in China are closely associated with drug trafficking routes [1], [18]. Like HIV, HCV can be transmitted by injection drug use (IDU), sexual contact and mother-to-child infection, and IDU is the predominant mode of HCV transmission in Asia [19], [20], [21], [22]. Our recent study [23] revealed that the HCV 3a strains circulating among Jiangsu IDUs were evolutionarily associated with the strains from Xinjiang IDUs, suggesting a potential geographic diffusion links between Xinjiang and Jiangsu, which is similar to the observation in HIV-1 CRF07_BC migration described here. Because of sharing a common transmission pattern (i.e. IDU), the coinfection with HIV-1 and HCV is very common among IDUs [24]. Therefore, we performed phylogeographic analysis using the *E1/E2* fragment of HCV 3a to test the potential association in migration between HCV 3a and HIV-1 CRF07_BC and CRF08_BC.

**Figure 4:**
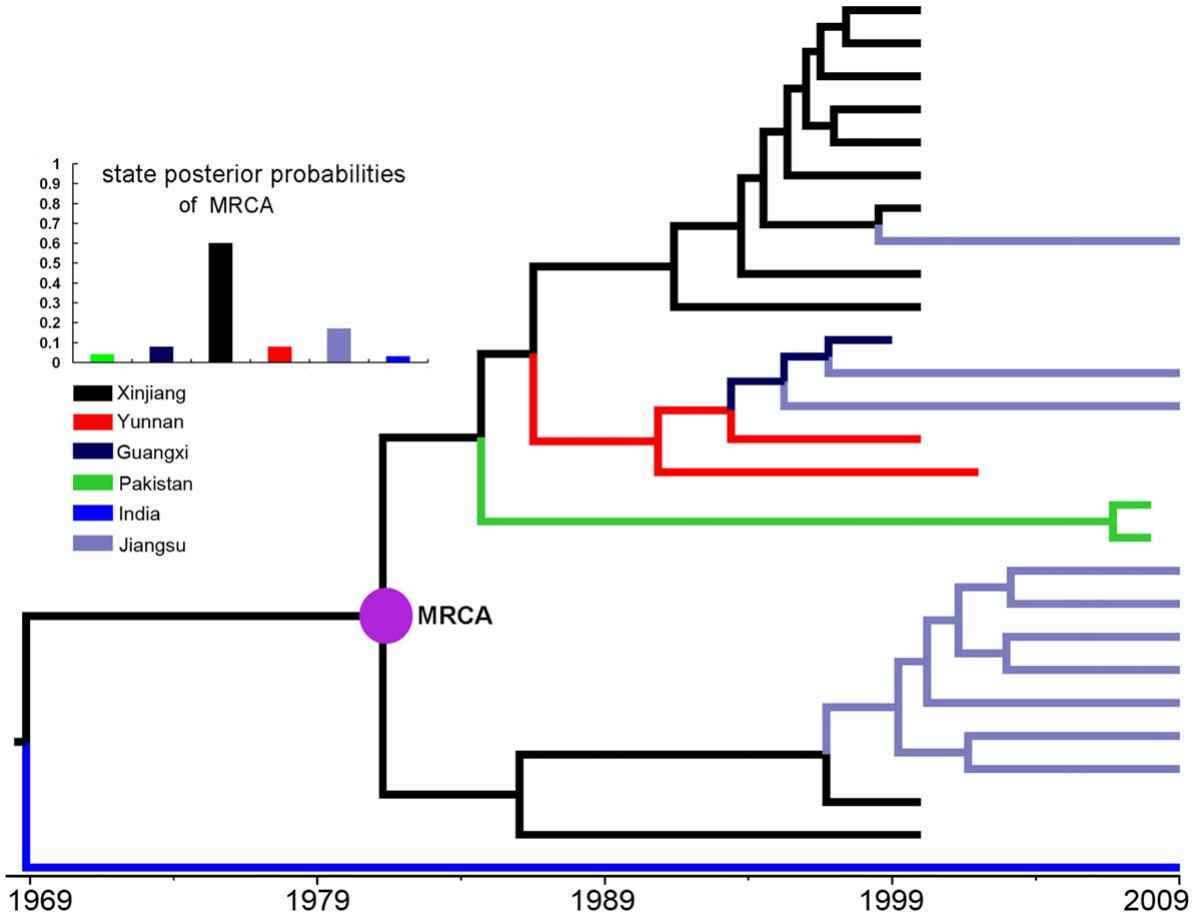
MCC tree of the HCV subtype 3a sequences. The tree was reconstructed base on the *E1/E2* region (H77 1356-1826 nt) of HCV subtype 3a. The state posterior probabilities of the most recent common ancestor (MRCA) are shown on the left upper panel.

The MCC tree shows that the HCV 3a strains from Jiangsu, Yunnan, and Guangxi clustered within the Xinjiang strains (Fig. 4). The ancestral state reconstruction indicates that Xinjiang was the geographic origin of the HCV 3a strains circulating in China (Fig. 4). The BF tests significantly support the geographic diffusion links between Xinjiang and Jiangsu (BF = 7.180) and between Yunnan and Guangxi (BF =  4.448) (Table 6). These results at least reveal two migration routes of HCV 3a from Xinjiang to Jiangsu and from Yunnan to Guangxi. In fact, the routes from Xinjiang to Jiangsu and from Yunnan to Guangxi were also observed on HIV-1 CRF07_BC and CRF08_BC migrations, respectively.

**Table 6:** The Bayes factors between defined locations of the HCV 3a *E1/E2* region.

Origin	Yunnan	Guangxi	Xinjiang
**Guangxi**	**4.448**		
**Xinjiang**	0.875	0.763	
**Jiangsu**	1.044	1.590	**7.180**

Bayes factors above 3 that represent statistically significant phylogeographic links between defined locations are shown in bold.

**Figure 5:**
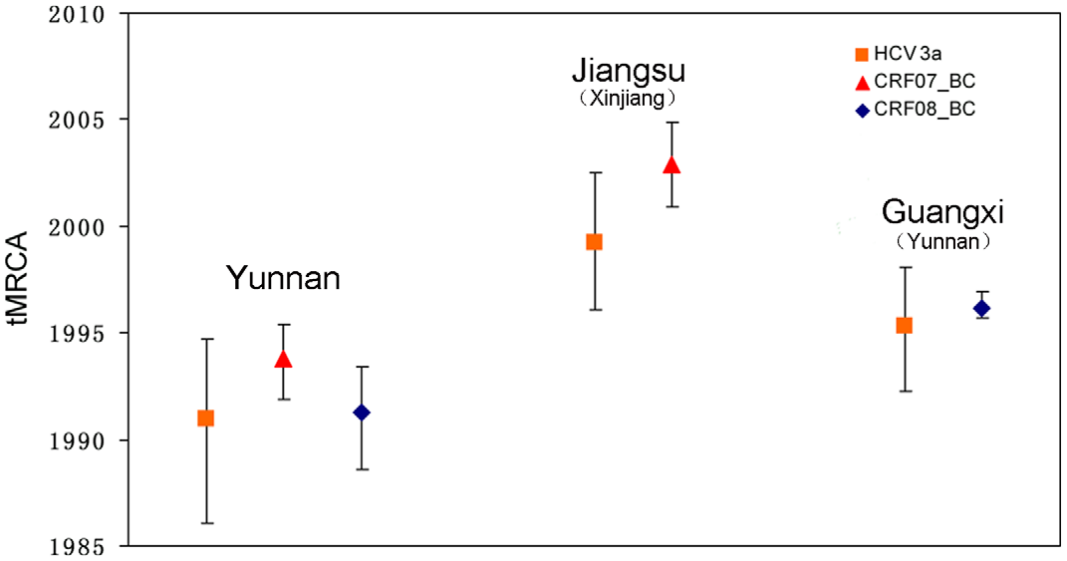
Times to the most recent common ancestors (tMRCAs) of HCV 3a, HIV-1 CRF07_BC and CRF08_BC from various geographic regions. The error bars represent the 95% credible regions of tMRCAs. The geographic origins of the strains from Jiangsu and Guangxi are shown in the parentheses.

In addition, we used BEAST package to estimate the evolutionary rate and the dates of tMRCA of HCV 3a. Since the genomic region of HCV 3a used in the Bayesian coalescence inference located in the hypervariable region, a high evolutionary rate of 7.157×10^−3^ substitutions/site/year was estimated under a constant size population model (Table 5). The estimated dates of tMRCA reveal that after being introduced into Xinjiang in 1981.3 (95% CR, 1973.1–1988.6), HCV 3a spread from Xinjiang to Yunnan in 1990.10 (95% CR, 1986.1–1994.7) and to Jiangsu in 1999.2 (95% CR, 1996.1–2002.5) (Fig. 5). From Yunnan, HCV 3a was further transmitted into Guangxi in 1995.3 (95% CR, 1992.3-1998.1). The dates of HCV 3a migration from Xinjiang to Jiangsu and from Yunnan to Guangxi were close to that occurred in HIV-1 CRF07_BC (2002.11, 95% CR, 2000.9–2004.11) and in CRF08_BC (1996.2, 95% CR, 1995.7-1996.9), respectively (Fig. 5).

## Discussion

Currently, China faces a rapidly increasing prevalence of two HIV-1 CRFs 07_BC and 08_BC [19], [25]. The investigation on temporal and spatial dynamics of both CRFs_BC may provide valuable information for HIV-1 epidemiology, prevention strategy, and vaccine development [12]. Because of the special importance of Yunnan in heroin smuggling and HIV-1 transmission [1], [2], [4], [20], almost all previous studies agreed that both CRFs_BC originated in Yunnan, and were transmitted to other regions of China via different drug-trafficking routes [11], [12], [13], [26].

**Figure 6:**
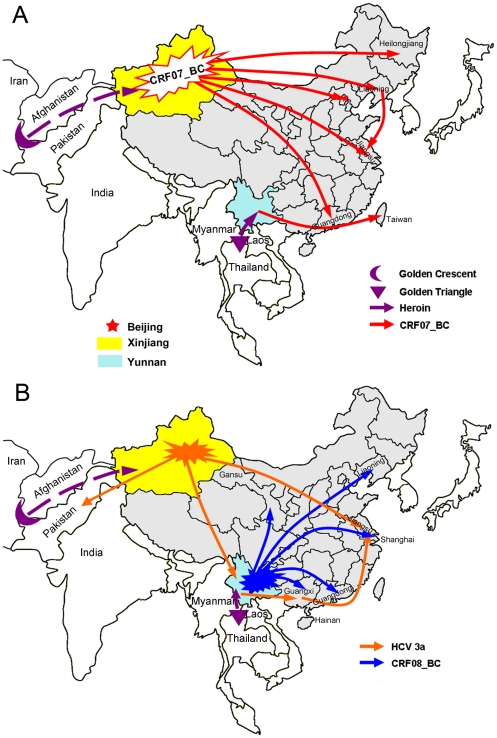
Plausible transmission routes of HIV-1 CRF07_BC, CRF08_BC and HCV 3a in China. (A) The transmission routes of CRF07_BC. (B) The transmission routes of CRF08_BC and HCV 3a.

In this study, we investigated the origin and phylogeography of HIV-1 CRF07_BC based on multiple genomic regions with more tip-dated sequences using Bayesian phylogeographic inference framework [14], [27]. Our results show that HIV-1 CRF07_BC spread to other regions (including Liaoning, Jiangsu, Heilongjiang, Beijing, and Guangdong) of mainland China from Xinjiang, and only the CRF07_BC circulating in Taiwan was from Yunnan (Fig. 6**A**). The dates of CRF07_BC spreading from Xinjiang to Liaoning and from Liaoning to Jiangsu were estimated at 1996 and 2000, respectively. Because HIV-1 CRF07_BC spread from Xinjiang to other regions (especially Liaoning) of mainland China, the two unanswerable scientific questions mentioned in the Introduction, namely why was only CRF07_BC transmitted northwestward to Xinjiang, but not CRF08_BC and why CRF07_BC was able to spread rapidly to the regions (incl. Xinjiang) far away from Yunnan so shortly after it originated are resolved. In addition, the analyses of the *gag* fragment of subtype C origin and the *gag-pol* fragment of subtype B' origin show that the geographic origin of CRF07_BC was Yunnan, whereas the analyses of the *env* fragment of subtype C origin suggest that Xinjiang was the origin region of CRF07_BC. These indicate that besides Yunnan, Xinjiang might also be the candidate of the geographic origin of CRF07_BC. In spite of this, the geographic origin of HIV-1 CRF07_BC still needs to be determined with more HIV-1 CRF07_BC strains from the regions (i.e. Gansu and Sichuan) between Xinjiang and Yunnan.

Although these results do not concur with Tee et al. [11], [12], in which CRF07_BC was considered to originate in Yunnan and spread from Yunnan to other regions of China (i.e. Xinjiang, Liaoning and Taiwan), we believe that our results are more reliable and accurate. First, the Bayesian method used in this study has been demonstrated to be more robust of describing the most plausible scenario of geographic migration of infectious disease than the model-free heuristic approaches implemented in PAUP* [14], although the latter was previously used to investigate the spatial dynamics of viruses, such as HIV-1 [28], HCV 1b [29], and influenza A H5N1 virus [30], [31]. Different from previous studies, in which the inferences of origin and phylogeography were only based on the phylogenetic trees [11], here we determined the origin and phylogeography of CRF07_BC and CRF08_BC via the reconstruction of ancestral geographic states and the BF tests. Second, the selection of the representative sequences is very important for phylogeographic study. In this study, we added more tip-dated CRF07_BC sequences representing a relatively wide range of locations across China into our analyses. These sequences include CRF07_BC strains sampled in the early CRF07_BC epidemic and a pure subtype C sequence isolated in the early HIV-1 epidemic in Ruili, Yunnan [7]. Third, in order to obtain a more comprehensive scenario of the geographic migrations of CRF07_BC and CRF08_BC, we selected multiple non-recombinant genomic regions (*gag*, *gag-pol* and *env*) of subtype C or B' origin to perform phylogeographic analyses, compared with the single genomic region to be used in the previous studies. Further, two loci of subtype C origin located in the *gag* or *gag-pol* region were merged into one fragment to enhance the accuracy of phylogenetic analyses.

We performed phylogeographic analyses of CRF08_BC using the same method. The results not only clearly confirm the previous conclusion that CRF08_BC originated from Yunnan in the early 1990s and spread to Guangxi and Liaoning in mid-1990s, but also show that the CRF08_BC isolates from Guangdong and Shanghai have close genetic relationships with the strains from Yunnan (Fig. 2**C** and 6**B**), further strengthening the importance of Yunnan in the transmission of CRF08_BC in China. Furthermore, we also analyzed the temporal and spatial dynamics of HCV 3a in Asia because it shares same transmission patterns with HIV-1 and is predominantly transmitted through IDU in Asia [19], [20]. The results show that HCV 3a was initially introduced into Xinjiang in 1981.3 and subsequently dispersed into Yunnan and Jiangsu in 1990.10 and 1999.2, respectively (Fig. 6**B**). Although HCV 3a and HIV-1 CRF07_BC emerged in Xinjiang at different times (1981 and 1993, respectively), both of them migrated to Jiangsu during a very short time interval (Fig. 5). In addition, the migration date of HCV 3a from Yunnan to Guangxi is also very close to that of HIV-1 CRF08_BC (Fig. 5). The similarities in temporal and spatial dynamics between HCV 3a and HIV-1 CRF07_BC and/or CRF08_BC suggest a possible association in migration patterns between different viruses through same transmission patterns (e.g. IDU). On the other hand, the fact that both HIV-1 CRF07_BC and HCV 3a were transmitted from Xinjiang to other regions of China indicates that besides Yunnan, Xinjiang also plays a crucial role in the transmission of viruses (at least HIV-1 CRF07_BC and HCV 3a) in Asia through IDU.

Xinjiang borders India and Pakistan and is near the ‘Golden Crescent’ region (another of the largest heroin-producing areas in the world). Like Yunnan, Xinjiang also possesses a geographic precondition to be an important entry point for heroin smuggling into China and other Asia countries or regions [2], [20]. However, people often associated the HIV-1 transmission among IDUs with overland heroin trafficking routes through Yunnan, and rarely recognized the role that Xinjiang plays in drug-trafficking and even the transmission of infectious viruses. The reason might be that Yunnan borders the ‘Golden Triangle’ region and in 1989, the first HIV-1 outbreak was detected among IDUs in Ruili city of Yunnan [4], [32]. From the MCC tree of HCV 3a, we found that two strains isolated from Pakistan in 2008 clustered within the Xinjiang clade (Fig. 4). This implies that there is a drug trafficking route linking Xinjiang and Pakistan (near the Golden Crescent), and along this route, HCV 3a was transmitted from Xinjiang to Pakistan. Furthermore, the phylogeography of HIV-1 CRF07_BC and HCV 3a clearly show that the HIV-1 CRF07_BC and HCV 3a strains circulating in other regions of mainland China were transmitted from Xinjiang, supporting the presence of multiple drug trafficking routes linking Xinjiang and other regions of mainland China. Therefore, as the most important transfer station for drug trafficking from Golden Crescent to China and other Asia countries or regions, the role of Xinjiang in transmission of infectious viruses such as HIV-1 and HCV in Asia should be especially considered.

## Materials and Methods

### Sequence data collection and pretreatment

HIV-1 CRF07_BC and CRF08_BC nucleotide sequences with known sampling years were downloaded from the Los Alamos HIV Sequence Database (www.hiv.lanl.gov). For phylogenetic and evolutionary analyses of CRF07_BC, three non-recombinant regions, including the *gag* fragments (HXB2 793-1211 nt and 1462-2061 nt) and the *env* fragment (HXB2 7095-7328 nt) of subtype C origin, and the *gag-pol* fragment (HXB2 2102-2601 nt) of subtype B' origin, were selected. In order to obtain more reliable results of CRF07_BC, the two *gag* fragments of subtype C origin were merged. The dataset of the merged *gag* region includes 36 CRF07_BC sequences isolated from the Chinese provinces of Xinjiang (n = 6), Yunnan (n = 10), Liaoning (n = 8), Jiangsu (n = 11), and Taiwan (n = 1). The dataset of the *gag-pol* region includes 37 CRF07_BC sequences isolated from the provinces of Xinjiang (n = 6), Yunnan (n = 10), Liaoning (n = 8), Jiangsu (n = 12), and Taiwan (n = 1). The Dataset of the *env* region includes 141 CRF07_BC sequences isolated from the Chinese provinces of Xinjiang (n = 105), Yunnan (n = 2), Jiangsu (n = 15), Beijing (n = 2), Guangdong (n = 4), Heilongjiang (n = 1), and Taiwan (n = 12). For CRF08_BC analyses, the *gag-pol* fragments (HXB2 794-1209 nt and 1709-2826 nt), the *pol* fragment (HXB2 2452-2844 nt) and the *env* fragment (HXB2 7095-7328 nt) of subtype C origin, and the *gag* fragment (HXB2 1258-1658 nt) of subtype B' origin, were selected. Similar to CRF07_BC analyses, the *gag-pol* fragment of CRF08_BC was obtained by merging the two discontinuous fragments of subtype C origin. The dataset of the merged *gag-pol* region includes 39 CRF08_BC sequences isolated from the Chinese provinces of Yunnan (n = 31), Gansu (n = 1), Guangxi (n = 3), and Liaoning (n = 4). The dataset of the *gag* fragment includes 41 CRF08_BC sequences isolated from the provinces of Yunnan (n = 31), Gansu (n = 1), Guangxi (n = 3), and Liaoning (n = 6). The dataset of the *pol* fragment includes 117 CRF08_BC sequences isolated from the Chinese provinces of Yunnan (n = 41), Guangxi (n = 36), Liaoning (n = 6), Shanghai (n = 6), Guangdong (n = 26), Gansu (n = 1), and Hainan (n = 1). The dataset of the *env* fragment includes 78 CRF08_BC sequences isolated from the provinces of Yunnan (n = 30), Gansu (n = 1), Guangxi (n = 46), and Hainan (n = 1).

Nine pure subtype C sequences from India, 1 from Myanmar and 1 from Ruili city of Yunnan province were used as the outgroup founder strains in the analyses of subtype C parts of CRF07_BC and CRF08_BC. Moreover, 9 pure subtype B' sequences from Thailand and 1 from Myanmar were used as the outgroup founder strains in the analyses of subtype B' part of CRF07_BC and CRF08_BC.

Hepatitis C virus (HCV) subtype 3a sequences located in *E1/E2* region (H77 1356-1826 nt) with known sampling years were downloaded from the Los Alamos HCV Sequence Database (www.hcv.lanl.gov). The HCV 3a dataset includes 2 sequences from Pakistan, 1 from India and 24 from China (Xinjiang, n = 11; Yunnan, n = 2; Guangxi, n = 1; Jiangsu, n = 10). The sequences of HIV-1 and HCV from Jiangsu have been previously reported by our laboratory [23], [33].

The datasets of HIV-1 CRF07_BC, CRF08_BC and HCV 3a in this study are available as Dataset S1, Dataset S2 and Dataset S3, respectively.

### Phylogenies and temporal dynamics analyses

All sequences were aligned using CLUSTAL W program implemented in MEGA 4.0 [34] and then manually edited. Maximum clade credibility (MCC) trees were constructed using a MCMC (Markov Chain Monte Carlo) method implemented in the BEAST v1.5.4 package, which incorporates time-of-sampling information and returns rooted trees [27]. All of these trees were viewed and edited using FigTree v1.3.1 (tree.bio.ed.ac.uk/software/figtree/). BEAST was also used to estimate the evolutionary rates and the dates to tMRCA of various nodes on the MCC tree [35]. A relaxed molecular clock with an uncorrelated log-normal distribution [36], a Hasegawa-Kishino-Yano (HKY) nucleotide substitution model [37] with a gamma-distributed model of among site rate variation using four rate categories (Γ4) [38], and a constant population size model were used in the Bayesian coalescence analyses [39]. Statistical uncertainty in parameter estimates was reflected by the values of the 95% highest posterior density (HPD) credible region (CR). Each MCMC analysis was run for at least 20 million generations, with sampling every 10,000 generations. The initial 25% of the samples were discarded as burn-in, leaving 1501 trees per run, when we summarized the trees using TreeAnnotator implemented in the BEAST v1.5.4 package. Posterior probabilities for the internal nodes were calculated from the posterior density of trees. The program Tracer v1.4.1 (tree.bio.ed.ac.uk/software/tracer/) was used to check for the convergence and to determine whether effective sample size (ESS) >200. The posterior densities were calculated with 10% burn-in using Tracer v1.4.1. If the effective sample size is less than 200, the MCMC chain length would be elongated to 200 million.

### Phylogeographic analyses

Each sequence was assigned a character state reflecting its sampling location firstly. The Bayesian phylogeographic inference framework was performed to analyze the strength of the movement between geographic locations using a geographically explicit Bayesian MCMC method implemented in the BEAST v1.5.4 package [14], [27]. This method can be used to infer the location state of the ancestral branch over the whole tree and to build a reversible diffusion rate matrix between previously defined locations accompanied with the evolutionary and coalescent parameters [14], [40], [41], [42], [43], [44], [45]. We set up a discrete phylogeographic analysis using a standard continuous-time Markov chain [46]. To investigate the phylogeographic diffusion process along the posterior sets of the trees, the relationships between these locations were identified using the Bayesian stochastic variable search selection. The Bayes factor (BF) test that determines the statistically significant phylogeographic links was performed using the RateIndicatorBF tool, which was recently added into the BEAST code [14]. If BF>3, the phylogeographic link between two locations was considered to be statistically significant [14]. Moreover, the posterior probabilities for the ancestral geographic states were also calculated from the posterior density of trees summarized by using TreeAnnotator implemented in the BEAST v1.5.4 package [27].

## Supporting Information

Figure S1 **The** ***env*** **MCC trees of HIV-1 CRF07_BC and CRF08_BC sequences.** Ancestral geographic states were reconstructed using Bayesian phylogeographic inference framework implemented in the BEAST v1.5.4 package. The tree branches are colored according to their respective geographical locations. The purple solid nodes on the trees represent the most recent common ancestor (MRCA) of CRF07_BC or CRF08_BC. (A) The MCC tree reconstructed based on the CRF07_BC *env* fragment (HXB2 7095-7328 nt) of subtype C origin sampled in and after 2000. (B) The MCC tree reconstructed based on the CRF08_BC *env* region of subtype C origin (HXB2 7095-7328 nt).
(DOC)

Dataset S1 **The datasets of HIV-1 CRF07_BC.**
(RAR)

Dataset S2 **The datasets of HIV-1 CRF08_BC.**
(RAR)

Dataset S3 **The dataset of HCV 3a.**
(RAR)
