# Supplementary material for: Phylogeographic Analyses Reveal a Crucial Role of Xinjiang in HIV-1 CRF07_BC and HCV 3a Transmissions in Asia
Source: PLoS One. 2011 Aug 12;6(8):e23347. doi: 10.1371/journal.pone.0023347 (PMC3155551; doi:10.1371/journal.pone.0023347)
Supplement: Figure S1 — The env MCC trees of HIV-1 CRF07_BC and CRF08_BC sequences. Ancestral geographic states were reconstructed using Bayesian phylogeographic inference framework implemented in the BEAST v1.5.4 package. The tree branches are colored according to their respective geographical locations. The purple solid nodes on the trees represent the most recent common ancestor (MRCA) of CRF07_BC or CRF08_BC. (A) The MCC tree reconstructed based on the CRF07_BC env fragment (HXB2 7095-7328 nt) of subtype C origin sampled in and after 2000. (B) The MCC tree reconstructed based on the CRF08_BC env region of subtype C origin (HXB2 7095-7328 nt). (DOC) [file pone.0023347.s001.doc]

**
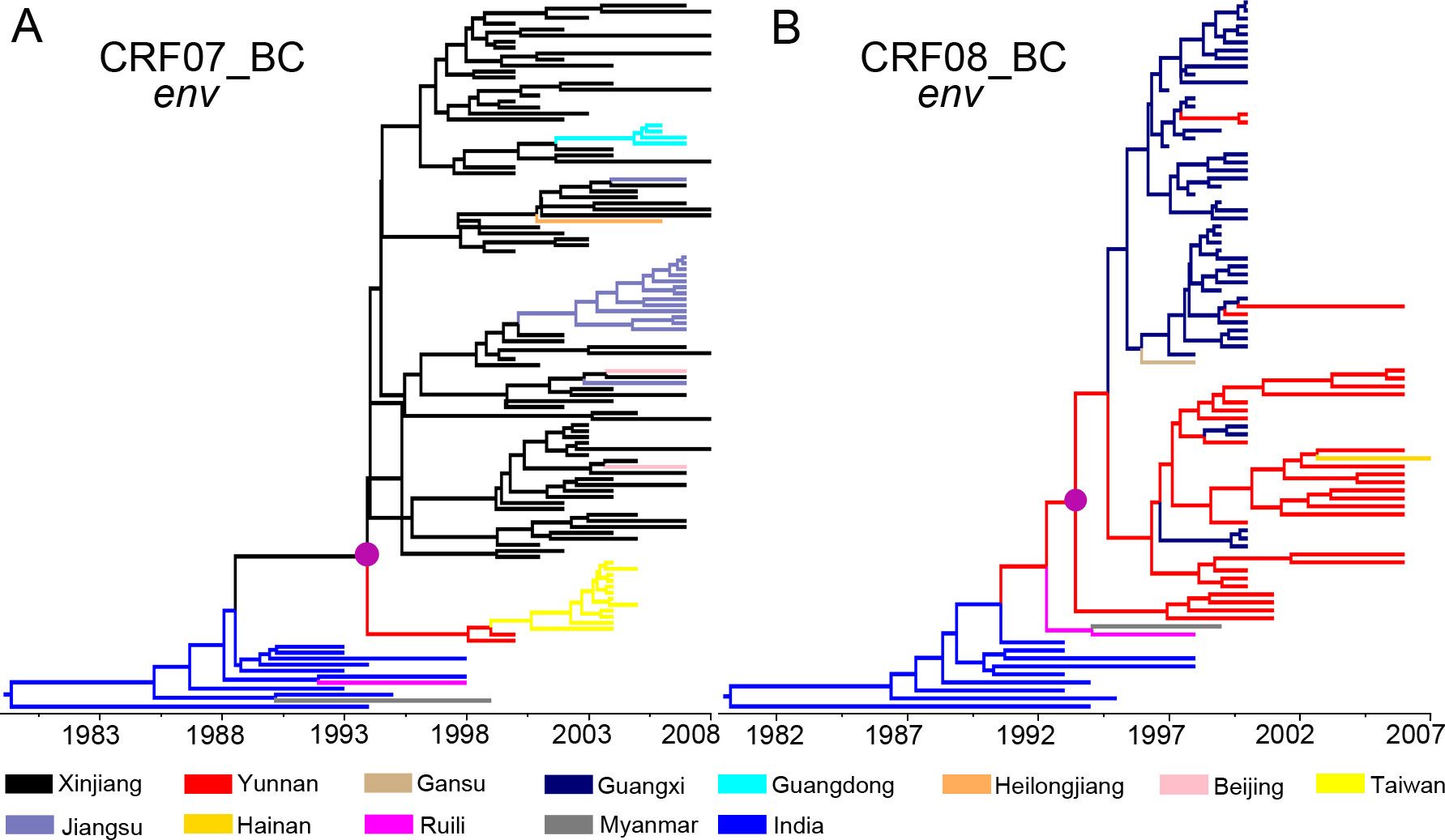
**

**Figure S1. The *env* MCC trees of HIV-1 CRF07_BC and CRF08_BC sequences.** Ancestral geographic states were reconstructed using Bayesian phylogeographic inference framework implemented in the BEAST v1.5.4 package. The tree branches are colored according to their respective geographical locations. The purple solid nodes on the trees represent the most recent common ancestor (MRCA) of CRF07_BC or CRF08_BC. (A) The MCC tree reconstructed based on the CRF07_BC *env* fragment (HXB2 7095-7328 nt) of subtype C origin sampled in and after 2000. (B) The MCC tree reconstructed based on the CRF08_BC *env* region of subtype C origin (HXB2 7095-7328 nt).
